# Self in Art/Self As Art: Museum Selfies As Identity Work

**DOI:** 10.3389/fpsyg.2017.00731

**Published:** 2017-05-09

**Authors:** Robert Kozinets, Ulrike Gretzel, Anja Dinhopl

**Affiliations:** ^1^Annenberg School of Communication and Journalism, University of Southern California, Los AngelesCA, USA; ^2^Laboratory for Intelligent Systems in Tourism, Los AngelesCA, USA

**Keywords:** selfie, museum, identity, embodiment, performance, aesthetic consumption, self-presentation

## Abstract

Selfies, digital images characterized by the desire to frame the self in a picture taken to be shared with an online audience, are important reflections of the contemporary self. Much extant psychological research on selfies has taken a pathologizing view of the phenomenon, focusing on its relationship to narcissism. Our investigation seeks to contribute to a holistic, contextualized and cultural perspective. We focus on the context of museums, places where art, history, education, and culture merge into the selfie taking behaviors of patrons. First, we explore theory salient to our topic of selfie taking, finding selfies to be an important way to construct ongoing series of narratives about the self. We use concepts of identity work, dramaturgy, and impression management to understand it in this light. We relate embodiment within the museum to the selfie’s performative acts and expand upon notions that emphasize and distinguish the aesthetic elements present in many aspects of everyday life. We also question the ability of the museum selfie to destabilize. We also explore the contextual effects of mimicry and social norms. After describing our ethnographic and netnographic method, we investigate the museum selfie phenomenon. We begin with some observations on the extent of selfie-taking in contemporary culture as well as its evolution. Then, we consider selfies as a type of dynamic art form. Our analysis identifies a range of different types of museum selfies: art interactions, blending into art, mirror selfies, silly/clever selfies, contemplative selfies, and iconic selfies. Considered and studied in context, the museum selfie phenomenon reveals far more than the narcissism of the sort explored by past psychological research. The museum provides a stage for identity work that offers an opportunity for the selfie to be used not only for superficial performances but also in the pursuit of more profound self-reflection and its communication. Our ethnographic exploration of the selfie sees it as more than a quest for attention but less than a genuinely destabilizing social force. Selfie taking is complex and multidimensional, a cultural and social act, a call for connection, an act of mimicry, and part of people’s ever-incomplete identity projects.

## Introduction

If our photographs are reflections of the way we see the world, selfies are reflections of the way we see ourselves. Yet they are more than mere self-reflection. They are intended for wider audiences, as if they were a form of art. As Iqani and Schroeder (2015, p. 408) explain, “an instructive starting point for thinking about the historical context of today’s selfie is the artistic self-portrait. In the West, self-portraits emerged as an important visual genre in and around the 16th century, typified by painters such as Albrecht Dürer and Rembrandt. These painters used self-portraiture to enshrine themselves as artists, as well as to reveal the inner depths of their character”. Carbon (2017) uses an art history perspective to explore this artful element of selfies. He finds that selfies aim to communicate and express complex, multidimensional cultural messages similar to those of self-portraits from the domain of artistic painting have done for centuries (see Schroeder, 2002, 2013). Selfies “reveal something about the creator in particular, but also something about humans in general” (Carbon, 2017, p. 17). This connection between photographs, art, communications, and the self are key elements of our investigation. Contemporary selfie taking is a complex, enculturated, and multidimensional phenomenon. To genuinely understand it in all of its complexity, the field must study it with a myriad of different investigative approaches. Hence, we contribute to this multidisciplinary discourse with a cultural approach and ethnographic methodology.

Selfies are public reflections of the way we view and present ourselves, an intriguing combination of inward and outward looking. Their pervasiveness has been facilitated not only by networked technology and devices such as front facing cameras and selfie sticks, but also by the internalized social conventions that make the capture and sharing of self images desirable and acceptable (Larsen and Sandbye, 2014). That these conventions are shifting is evidenced by the changing and amorphous definitions of what constitutes a selfie (Hess, 2015; Senft and Baym, 2015). Sorokowski et al. (2015, p. 124) define selfies as photographs “of oneself (or of oneself and other people), taken with a camera or a camera phone held at arm’s length or pointed at a mirror, that [are] usually shared through social media”. This fundamental notion captures the core elements of the selfie phenomenon. However, that phenomenon is constantly changing as the practice evolves. Some literature now adopts a broader definition to accommodate group selfies, partial selfies of body parts, timers, selfie sticks, and highly manipulated photos facilitated by app technologies such as Snapchat. Rather than confining the selfie phenomenon to a particular technology or genre of photograph or video, we follow the broad definition of Dinhopl and Gretzel (2016, p. 127), which identifies selfies as “characterized by the desire to frame the self in a picture taken to be shared with an online audience”.

Psychological research on selfies has emerged as a vital and growing sub-field. Psychology research has explored motivations for selfie-posting (Pounders et al., 2016; Sung et al., 2016), age and gender differences in posting selfies (Dhir et al., 2016), and self-esteem based effects of selfie posting (Wang et al., 2017). Much of this research has frequently taken a pathologizing perspective on the phenomenon, focusing on the relationship between narcissism and posting selfies (e.g., Fox and Rooney, 2015; Lee and Sung, 2016). In particular, by focusing on extremes of high selfie posting behavior and viewing the activity in an excessive, individualistic, and decontextualized manner, psychological research may be obscuring some of the most interesting aspects of the phenomenon. Indeed, like Internet and social media consumption itself, psychology research has linked selfie production to shallow relationships, lack of intimacy, loneliness, anorexia, risks to mental health, and a general lack of mental well-being (Adamkolo and Elmi-Nur, 2015, pp. 22–24).

Recent psychological research has begun to offer more nuanced views of the selfie phenomenon. For example, Sorokowski et al. (2015, p. 125) find that the measures of narcissism are “significantly and positively correlated with” the posting of selfies on social media sites, and also that the “link between narcissism and selfie posting is stronger among men than women”. However, another study found that posting selfies is a fairly common practice on social media sites, becoming “a typical way of communicating with others” and generally not related to narcissism (Barry et al., 2017, p. 7). Qiu et al. (2015) picture-coding scheme for selfies presents a psychological framework for image content analysis of selfies. The authors included facial expressions and position of the self as variables and categorized location as public or private. Their study emphasized the importance of context for representation in selfies. These results, along with the shifting definition of selfies, point to a dynamic and complex phenomenon which is increasingly embedded in contemporary communications.

We believe that an alternative approach would be useful in recontextualizing selfie taking away from pathologies and toward an alternative view. Relevant to this view are historical approaches such as those of Schroeder (2002, 2013), Iqani and Schroeder (2015), and Carbon (2017). The view we propose is that selfie taking is a complex and multidimensional phenomenon embedded in a wider set of evolving contemporary social practices. Drawing inspiration from the work of Rounds (2006) and Burness (2016), we chose to examine selfie taking in the context of contemporary art museums.

Museums, obviously, are sites in which art, culture, and photography have a long history. Museums are also, it turns out, important sites of “identity work” which encompasses the psychological “processes through which we construct, maintain, and adapt our sense of personal identity, and persuade other people to believe in that identity” (Rounds, 2006, p. 133; see also Howard, 2000). Museums have been found, perhaps unsurprisingly, to be an increasingly important site for selfie taking behavior. For instance, Blühm (2016) discusses how the management of the Groninger Museum in the Netherlands has been altered by social media and the rise of selfie taking. Burness (2016, p. 95) provides an extensive overview of the museum selfie phenomenon, and quotes poet laureate Ken Goldsmith as saying that the Mona Lisa and other iconic artworks have become “wallpaper for selfies”. She finds the moral panic surrounding potential damage to artwork from careless selfie takers at museums to be prevalent. Given the aforementioned importance of the selfie’s connections to art and also the need for a more contextualized understanding of a phenomenon that has been largely studied as the isolated and decontextualized behavior of narcissistic individuals, we find the museum setting ideal for an identity work-focused investigation of the selfie.

By locating it in a specific public place, one that combines history, education, and culture, we embed our understanding of selfie taking in a broader, more cultural, and more social direction than past psychological research. Respectfully extending the museum scholarship emphasis of Rounds (2006) and Burness (2016), we bring a deepened psychological perspective to the contextualized phenomenon of the museum selfie. We begin our investigation with a look at several relevant theories. First, we extend a Lacanian “mirror stage theory” perspective (Lacan, 1977), taking its interlinked notions of self image, maturity and visual development in a technological direction. We consider whether the hall of mirror effects of our devices might reveal something about the regressive possibilities of contemporary adult identity. Does selfie taking, studied in context, act merely to elevate the self, or to provide a complex amalgam of self and setting, as Dinhopl and Gretzel (2016) suggest in their contextualized study of travel related selfie taking? In summary, our perspective seeks a cultural viewpoint on selfie taking, considering it to be a set of social practices intimately linked to the most intimate of pursuits: identity work.

Psychological impression management theory posits that people are inclined to create and share impressions of themselves which are biased in the direction of their desired identities (Markus and Nurius, 1986). Similarly, the sociologist Goffman (1959) emphasized the importance of self-presentation strategies to control impressions of the self, often also highlighting the role of factors and contexts external to the individual. Schau and Gilly (2003), Belk (2013) and a range of other researchers have described and analyzed the self-presentation related motives that individuals bring to their digital communications. Murray (2015) portrays selfies as effective outlets of self-definition, creative forms of self-fashioning, and therefore powerful means of self-expression. In Belk’s (1988, 2013) “extended self” perspective, selfies represent digital possessions that play an important role in establishing and signaling identity. A recent investigation by Pounders et al. (2016) found that “consumers were motivated to post-selfies to convey a positive self-image”. Findings from this study also revealed that desired images included looking happy, having fun and projecting a positive physical appearance. Our identity work investigation complicates this perspective. What messages are conveyed by selfies taken with art? Is this mere happiness and beauty, or might it be something more? More importantly, what are the social and cultural contexts in which selfie taking in general might be more productively viewed?

The paper proceeds as follows. First, we explore theory salient to our topic. We examine theory linking identity and consumption, museums and embodiment, the aesthetics of consumption, performance and staging, and mimicry and social norms. Then, we offer some elaboration of our ethnographic and netnographic approach to the contextualized study of museum selfies. The next section presents our findings, which center upon a categorization of the different types of selfies we observe being taken in art museum contexts. Finally, we offer a concluding section that discusses the implications of our findings. Our results may have useful implications to help psychologists and others scholars of the selfie develop a more multifaceted view of selfies than is exhibited in the current pathologizing literature.

## Literature Review

The dichotomized view of selfies as authentic expressions of identity and self-absorbed distortions persists throughout most of the scholarship on the topic. Carbon (2017) positions selfies as artifacts in a long history of self-portraits in art while Wendt (2014) more cynically sees them as parodies of portraits in the social media age, exemplified by artificial poses such as “duck face”. Jones (2002) argues that the selfie is an inflated performance of the self. Similarly, Levin (2014, p. 20) describes selfies as “portraits of the self in the act of self-portrayal,” emphasizing the practice rather than the outcome. Wendt (2014) emphasizes that visual social media encourage selfie-taking, animating users to create infinite versions of themselves through selfies, and keep users continuously engaged with images of themselves. Others conceptualize selfies as means of communication that afford a transformation of a personal experience into a shared one (Molz, 2012).

What does seem clear is that selfies are a means by which individuals can insert images of themselves into communications in a way never before possible. Our discussion of selfies is thus influenced by a perspective that views them as communicative aspects not only of individual identity, but of individual aspects of the networked self (Papacharissi, 2010). Online selves influence one another. They are ever more carefully curated (e.g., social media users delete posts that do not receive the desired number of likes in order to not taint their social media identity), which spurs a quest for the extraordinary and high scrutiny of what is share-worthy (Dinhopl and Gretzel, 2016). Rettberg (2014, p. 35) offers the most fully realized view of this highly interconnected and highly contextualized sense of the selfie. Her work connects the selfies with visual identity, time lapse photography, and changes in digital profile pictures to argue for an embedded view in which “digital self-presentation and self-reflection is cumulative” and part of an ever-evolving progressive series. This view accords well with the identity work and art emphasis of the museum selfie.

### Identity Work Involving Objects and Places

Identity emerges from “a dialectic between internal identification and external ascription” (Howard, 2000, p. 375). It is the mediating function between what is inside the self and what is outside, between the agent who chooses to act and the structures that provide the opportunities for acting, alternatives among which actions may be chosen, and the consequences of acting. Our identity work, the generator of our individuality, “is not so much a state to be achieved as a mode of life to be pursued” (Appiah, 2006, p. 5). Identity work, like serial selfie taking, is processual. Identities and profile pictures always exist as works in progress.

Consumer culture theoreticians Ustuner and Holt (2007, p. 51) use the term “identity project” to refer to the self-narratives that people form as they continuously develop their identities by projecting “the constructed past into the imagined future”. According to them, people selectively update the narratives they tell themselves and others about their lives and who they are “as they interpret and incorporate the real twists and turns [that occur] as their lives progress” (p. 51). This continuous act of story building and storytelling seems highly salient to our contemporary culture (Mick and Buhl, 1992). Cultural communications such as art, fashion, and advertising symbols are used as sources of ‘symbolic resources’ by people, who seek in them “new ideas and better concrete versions of old ideas with which to advance their [identity] project” (McCracken, 1987, p. 122). In this research, we build on the notion, also developed by Kozinets et al. (2004) that physical spaces and objects are also used as symbolic resources in contemporary identity projects. As Burness (2016, p. 115) states, museum selfies “point to the social role that objects play in the lives of our visitors and the important role that museums have in facilitating those relationships”.

Rounds (2006) suggests that visiting a museum can simultaneously serve both the construction and signaling of identity. Museums allow visitors to associate their self with something that is larger than themselves. According to Rounds, museum visitors can transfer the special aura of importance that the displayed objects hold onto themselves. Hromack (2014) makes a more traditional possession-related and “extended self” type argument (cf. Belk, 1988), claiming that selfies are a gesture of ownership, a way of owning the art through its image. Selfies allow individuals to weave museum objects into their own identity. Foster (2014, p. 4) states that “taking a good selfie requires that both the creator/subject and the viewer look carefully at the artwork or artifact, granting a new perspective and a personal connection to a potentially lifeless object”. Also, museums as physical and cultural locations provide opportunities to both confirm existing identities and explore alternative selves; they are spaces of identity enactment. Mukherjee et al. (2015) describe museum spaces as offering psychological affordances that facilitate the directing, shaping, scaffolding and (re)producing of the psyche. Falk (2006, p. 151) also underlines the deep connection between identity and museum visitor experiences, basing his contextual viewpoint on the multiple selves view that “all individuals enact multiple identities, many of which are situational and constructed in response to a social and physical context”. Pekarik et al. (1999) highlight the introspective quality of museum experiences and therefore the opportunity to reflect not only on what is seen but also on one’s identity in relation to the museum displays.

Selfies often contain props. Brand-related selfies are “a nodal point” where the consumer’s own attempt to create their identity and share its positive impressions meet the corporate interest in managing the official impressions of the brand (Rokka and Canniford, 2016). This intersection potentially destabilizes both consumer-generated and corporate brand impressions (Hess, 2015). In their study, which centered upon people’s sharing of selfies with champagne brands, Rokka and Canniford (2016) find that the framing and tone of brand-related selfies resemble consumer generated advertising. People put themselves and the brand together. In this case, they borrow from the champagne brand’s meanings, but also, by sharing it with their network of friends and followers, they lend it social meanings of their own.

Commercial impressions of corporately controlled brands might be quite different from cultural impressions of ostensibly civic artistic works. We might ask if the combination of museum, person, art, and selfie is also a destabilizing force. Does it destabilize the artwork by publicizing it, reproducing it, cheapening it, and reducing its ineffable Benjaminian aura? Does it destabilize personal identity, and perhaps also the innately narcissistic tendency of the selfie practice? Does it do this by moving the focus away from the person, the expression, and the present time to the museum setting, the art work, the cultural legacy, and historical time? Rounds (2006) and Burness (2016) seem to conclude with a sanguine view that museum selfies are stabilizing forms of self-expression inspired by museum objects, a means of discovering and asserting one’s sense of self. In this paper, we will explore further these conflicting notions of psychological destabilization and stabilization. We will investigate not only if museum selfies are a destabilizing force, but also whether or not the frame of destabilization holds up to scrutiny. Throughout, we will interrogate whether museum selfies signal something about the meaning and purpose of being at the museum. Do they reveal something significant about art, contemporary identity, and the experience that brings the two together?

### Museums and Embodiment

Identity is implicitly linked with the body and, in this study, we explore the concept of lived embodied experience in museums. The study of embodiment, the combined conscious and unconscious sense of being physically present as a body in a particular space, has gained ground as an important concept in the social scientific study of experience (Lakoff and Johnson, 1999). The role of the body in lived experience seems central to the understanding of selfies. For instance, Adamkolo and Elmi-Nur (2015, p. 21) found that women tilted their head by 150% more than men in their shared selfies, a result which they related both to gender imbalance as well as to expressiveness, sexual provocativeness, and “moral decadence and abuse”.

In museum selfie research, studying the position of the body in regards to artwork has been an important concern (Larson, 2014; Burness, 2016). The museum, in this literature, is a type of consumption experience in which the entire body moves through space. As Falk (2006, p. 126) avers, the museum “visitor is maintaining or building identity” by being “actively engaged in using the social and physical context of the museum to make personal meaning”. Past research finds a strong relationship between museum visitation and notions of embodiment (Joy and Sherry, 2003; Burness, 2016). Museum goers “succumb to experiences” (Joy and Sherry, 2003, p. 261) in which the museum and its art act as “a background” and “a stage for what is seen” (ibid: 264).

In our research, museums act as stages for the entire embodied self, rather than merely being sites subjected to the visitor gaze. Museum experiences and selfies both are highly personalized, embodied and felt practices (Warfield, 2014; Burness, 2016). Taking selfies is a personalized way of moving through the museum. This personal, social, and physical meaning-making is an important focus of our investigation.

### Consumption of Aesthetics

Another way to think about selfie taking in the museum is to relate it to the consumption of aesthetics. Leder et al.’s (2004, p. 489) perspective on art is that the “cognitive processing of art produces affective, often positive and self-rewarding aesthetic experiences”. Aesthetics, the philosophies of art and beauty, are a school of philosophy dealing with concepts of order, harmony, and beauty in the material world. What these philosophies have in common is “the idea that aesthetic experience is central to a life of higher order; that is, aesthetic experience is distinguished from the material aspects of life and privileged because of its importance in human development” (Venkatesh and Meamber, 2006, p. 20). In this case, selfie taking in the museum could be understood as an aesthetic pursuit, perhaps relating to the ability of art’s harmonious visual properties to enhance self images and self expressions of those who utilize them. Alternatively, perhaps the museum selfie acts out an intention to disrupt or destabilize the stodgy museum setting and ossified artwork by turning it into a mundane backdrop, thereby adding a level of contemporary disharmony to art’s more balanced visual impression.

These notions of harmony and disharmony as well as stabilization and destabilization build upon the notion that there are aesthetic elements present throughout many of the ostensibly mundane aspects of everyday life. Historically, people’s participation in the arts was not perceived as an element of their ordinary day-to-day existence. However, Venkatesh and Meamber (2006), following many eminent scholars have suggested that art and everyday life are interlinked. Art, these scholars assert, has wrongly become a privileged term. In fact, “the artificiality of the separation between ‘high art’ and popular culture” has become increasingly apparent “as global media and information technologies accelerate the correspondence between the domains of art, popular culture, and commerce” (Venkatesh and Meamber, 2006, p. 24). Not only do everyday experiences have aesthetic qualities, but we can also conceive of aesthetics as playing a critical role in the creation and expression of contemporary personal identity (Venkatesh and Meamber, 2008; Schroeder, 2013; Iqani and Schroeder, 2015; Carbon, 2017), and thus as a cultural element, a defining “code” of current identity projects (Rounds, 2006). Our investigation seeks to broaden and develop Venkatesh and Meamber’s (2008, pp. 51–52) conception of people as “aesthetic subjects” who view not only everyday experiences and artistic products in an aesthetic sense, but who fluidly transfer aesthetic impressions from artistic surroundings to their own captured photographs of themselves. It also seeks to understand the viewpoint and implications of the creative and artistic notion that people become the “aesthetic objects” of their own selfies.

### Performance and Staging

Following the dramaturgical framing of Goffman (1959, p. 62), who noted the value of “dramatically inflated actions” such as museum selfie taking, we can productively view the phenomenon as a type of performance that occurs upon a particular sort of stage. Placing deliberate staging at the center of their typology of selfies, Presi et al. (2016) separate brand selfies based on the level of staging. Past research on museums finds many instances of visitors using the physical surroundings as a place of performance (Joy and Sherry, 2003; Kozinets et al., 2004; Hollenbeck et al., 2008). Prompted by cues in the built environment around them, individuals visiting these spaces strike poses in the act of playing and in the sense of informal performing for one another (Kozinets et al., 2002, 2004; Joy and Sherry, 2003).

In museums and in selfies, people perform their identities before one another as cultured, cultural beings (Falk, 2006; Rounds, 2006). With his notion of “enactments,” Rounds (2006) combines notions of embodiment and identity with the idea of museum consumption. The museum, he says, “offers a perfect setting for public performance of identity. It is a space designed for the display and performance of meaning. Visitors take advantage of that character to enact their own identities, borrowing for those identities a bit of the aura of special importance held by the objects on display” (Rounds, 2006, p. 142).

Larson (2014) describes how the *Sugar Baby* art installation in Brooklyn is utilized by visitors and their cellphone cameras. The 75-foot tall white female sphinx with a “Jemina-like face” and massive exposed breasts (p. 505) provided an almost irresistible backdrop for photographic engagement, and specifically selfie taking. Reflecting on the complex motivations for such observed behaviors by “fashionable young women, black and white” (p. 506), Larson (2014) writes that there is “some deep human trait…some need to insert ‘the self’ into every situation” (p. 511). She concludes that the art work stands somehow above this effrontery and intrusion: “She is bigger than life. She has absorbed every insult that has come her way and has transcended it” (ibid). The disruptive social transgressions and disruptions inherent in the performance of the artist, the art, and the art viewer have powerful psychological implications for our understanding of the phenomenon. In our research, we seek to study how people perform with art and capture their performance for particular purposes.

### Mimicry and Social Norms

A closer examination of the contextual effects of being in museums holds an important place in understanding selfie taking behavior. As Dimberg et al. (2000) note, mimicry is an unconscious and automatic process that is subjectively experienced as too strong for suppression. Chartrand and Bargh (1999) show that mimicry has strong adaptive effects: it creates liking and help to achieve an affiliation objective. Using the term “Chameleon effect” to refer to “non-conscious mimicry of postures, mannerisms, facial expressions, and other behaviors of one’s interaction partners….,” Chartrand and Bargh (1999, p. 893) see mimicry as an essential and important part of human social existence. However, Bourgeois and Hess (2008) find that mimicry does not require close relationships with interaction partners and, in fact that it can act as a powerful social cue and a signaling behavior.

Selfies are not only a social artifact but also a social practice (Senft and Baym, 2015). In the museum setting, parts of the surroundings are structural and aesthetic, such as the building and the collection of art, and part of the situation is composed of the social surroundings, which, as Belk (1975, p. 159) elaborates, constitute “other persons present, their characteristics, their apparent roles, and interpersonal interactions”. In the museum setting, these social surroundings might also include the sociotechnical aspects of other museum visitors taking selfies. In our research, we explore the embedded nature of selfie taking as a natural way in which people “do museums”—and much else. In response to this radical change, museum policies are moving toward providing more participatory experiences to accommodate the social trend of selfie-taking (Johnson et al., 2015). In our investigation, we inquire about the collective behavior of selfie taking in museums, particularly around certain exhibits or in certain places. We examine our field sites as situations or surroundings, and look for evidence of mimicry and for the establishment of contagious social norms of selfie taking practice.

## Methodology

Cultural psychology has long had “an affinity” with ethnographic methods based in a combination of participation and observation (Miller et al., 2003). Because our study is interested in a multidimensional, dynamic, complex, and contextual understanding of the selfie phenomenon, we found the use of ethnographic methods entirely appropriate to our psychological investigation. We used ethnographic methods to collect data on museum visitors and their selfies in North America, Europe and South America, and extended this approach into the online realm with the method of netnography. We briefly describe these approaches and their use in our study in the following section.

### Ethnography and Netnography

Ethnography is the established and venerable technique of cultural investigation which originated in the field of anthropology. Netnography is a specific adaptation of ethnography designed to maintain ethnography’s cultural approach and apply it to the study of online social interactions and experiences (Kozinets, 2015). Found useful in a range of studies in social sciences fields (Bengry-Howell et al., 2011), including psychology (e.g., Orsolini et al., 2015) netnography “links to a human consciousness project most closely aligned with gestalt psychology, cyber-psychology, and the anthropology of consciousness” (Kozinets, 2017, p. 382). Netnography adds novel procedures and research practices to the traditional routines of anthropology (Kozinets, 2002) that include locating sites and topics using search engines and handling large digital datasets with a combination of automated and manual techniques (Kozinets, 2015).

As qualitative research applied to questions of cultural psychology, ethnography and netnography help us to focus on data and analysis showing how psychological and behavioral tendencies can be understood through a deep investigation of their cultural underpinnings. Applied to a psychological phenomenon like museum selfie taking, ethnography and netnography reveal contexts of art, culture, expression, and self-representation that link to wider cultural phenomena such as media, technology, and fashion that influence the manifest behaviors. It therefore combines micro and macro-level collection and analysis of data in a study which shows the inseparability and co-constitution of selves, identities, identity projects, social networks, and cultures.

### Data

We focused our ethnographic observations on art museums as the most visually oriented genre of museums. Prolonged and deep as well as online and offline engagement with the phenomenon served as the key determinants of our selected research approaches. We not only collected primary data but also immersed ourselves in media accounts of selfie-taking in museums. Offline participant observation involved visits to the Broad Museum and LACMA in Los Angeles, California, the Pace Art and Technology Gallery in Palo Alto, California, and the Inhotim Museum in Brazil during 2016. During these visits the researchers observed selfie-taking at the museums and engaged in selfie-taking themselves, seeking out popular selfie spots within the museums that had been identified through online searches as well as prior observations at the museums. We shared these selfie images on our social networks, and kept field observations and reflective notes regarding their consequences, thus deepening our understanding of the internal processes and motivations of the behavior.

The online data collection efforts were focused on the social media platform Instagram because of its visual focus, posting to a public domain and extensive use of searchable hashtags. Searches were conducted in October 2016 and encompassed searching for related hashtags as well as location tags for particular museums. The search started with the general #museumselfie hashtag (29,139 posts) and continued with the museums that were included in the participant observation component of the research (#broadmuseum, 20,279 posts; #LACMA, #532,061 posts, #inhotim, 144,346 posts; #pacegallery, 27,863 posts as well as the respective location tags for these museums). The search was then expanded to include two museums that were prominently featured in the #museumselfie posts: the Louvre and the Musée D’Orsay in Paris (#louvremuseum, 136,853 posts; museedulouvre, 167,107 posts; #museedorsay, 98,584 posts; #dorsaymuseum, 1,136 posts). In recognition of the prominence of selfie-taking in connection with specific museum objects/exhibitions, the search also included #monalisa (504,733 posts) and #infinityroom (22,236 posts). Screenshots were taken of those posts that were images of the self (they had to include at least parts of the body in evidence). These screenshots included the hashtags and photo description as well as the visible comments. For each search, the most recent posts (up to 1 week prior to the search date) were investigated. In its entirety, our data set consisted of our observations, photographs, field notes, reflexive notes, downloaded photographs and screenshots.

### Coding and Analysis

The research followed a hermeneutic interpretive approach aimed at identifying emerging themes by iteratively circling back and forth between data and interpretation, from site to text. Visual and other semiotic data analysis techniques (Kozinets, 2015) were used on the corpus of fieldnotes, images that were created and collected, as well as on the textual descriptions of the Instagram posts.

#### Findings

We begin with some observations on the extent of selfie-taking in consumer culture as well as its evolution. Google Trends shows that selfie as a search term emerged in December 2012. Instagram currently features 277,724,072 posts that are tagged with the hashtag “#selfie”. That museums play an important role in facilitating selfie-taking becomes apparent through the extensive use of the selfie subcategory hashtags “#artselfie” (36,426 posts on Instagram) and #museumselfie (29,139 posts). Google Trends indicates that museumselfie as a search term emerged much later than the general selfie term, namely in January 2014. This was likely spurred by the creation of the first Museum Selfie Day in January 2014, an annual online event in which many museums participate and that encourages individuals to post-selfies taken in museums on Twitter or Instagram. This shows that selfie-taking in museums is not only a widespread but also a persistent phenomenon that engages 1000s of individuals on Instagram alone. The significance of the selfie-taking in museums phenomenon to practitioners is reflected in curatorial museum scholarship such as by Larson (2014) and Burness (2016). On a pragmatic level, it is apparent in the so-called “selfie museums” in Southeast Asia that present art objects especially selected for their suitability as selfie backgrounds (Nationalpost.com, 2015).

That selfies serve as important digital possessions for the extended digital and networked self becomes evident through the existence of the basic museum selfie that portrays the face of the person in front of museum objects/art. The museum object is clearly delineated from the person and often appears in its entirety in the picture. Significance is seemingly transferred to the self through proximity, and both art object and personal image are prominently featured. What is important is that this selfie communicates a very intimate, personal relationship with the art. No other museum visitors are visible and the descriptions often read something like “Vincent and me”. These selfies tangibilize the museum experience and make it possible to ascribe a fleeting moment in time visibly and irrevocably to one’s self. Because they are uploaded to the publicly accessible Instagram platform, they are not only a digital possession but also serve as an important piece in the online narrative about the self which must be read in the entire context of a person’s posting behavior on the medium to be fully comprehended in context.

Beyond this expected, general museum selfie type, which did not occur as frequently as we were expecting, the data analysis and observations revealed a multitude of other categories that contribute to identity projects in different ways. We describe and explain them in the following sections.

### Selfies As Art

Selfie-taking as an aesthetic consumption experience and the self as an aesthetic object come into play in a variety of ways in the selfies taken at museums. First, we find evidence for the “art as wallpaper” selfie as suggested by Goldsmith in Burness (2016). In these selfies, fragments of artwork form the background for close-up views of the self. The art serves one purpose only: beautification of the self that is portrayed in the selfie. Abstract art and big installations, as for example prominently displayed in the Inhotim museum in Brazil, lend themselves particularly well to these kinds of selfie projects, and the purpose of art as art (rather than as stage or backdrop) appears destabilized. Second, we identified selfies that strive to be artistic and therefore identify the selfie-taker as an artist. These selfies are different from others in that they play with light, camera angles and unique perspectives, and echo more closely than most other analyzed selfies the standards of self portraiture described by Carbon (2017). The selves portrayed in these pictures often strike an artistic-looking pose, such as looking off meaningfully into the distance, or with the hand gesticulating, touching the face or the chin. Rarely do the creators of these more artistic selfies look directly into the camera, as is the conventional practice. Instead, the subjects of these selfies are deliberately posed and framed to seem more like traditional portraits.

### Performances of the Self in Museums

Several types of selfies emerged from the data that pertain to two types of performances of the self. First, there is the embodied person performing for the camera (and for physically present other persons) in the museum context. Second, there are extended performances of the self that are shared in online communication spaces, and manifest through additional performance details such as captions, titles, comments, and hashtags. One selfie genre relates to interactions with the art. Poses held by statues or figures in paintings are replicated by the selfie-takers, sometimes pretending to touch the art—a practice that, as Burness (2016) emphasizes, is strictly forbidden and widely feared by museum curators and staff.

Blending into the art is another specific sub-genre facilitated by the exhibits. For example, inserting oneself into projected images at the Pace Gallery allows the art to appear on one’s body/face. The Infinity Room at the Broad Museum as well as the Urban Light installation at LACMA and several other smaller exhibits allow visitors to locate themselves as physically present inside the art installation or art work. Being located inside the art seems to encourage additional performances and trigger the need to see and show oneself performing, which is satisfied through selfie-taking and sharing. One of the selfies we found in this sub-genre had a poignant descriptor: “We are part of the art”.

Another type of selfie that fits within this group is the mirror selfie. Mirrored objects in museums (whether they are curated museum objects or simply reflective surfaces such as polished glass tables) act as magnets that attract selfie-taking performers. These visual watering holes bring museum visitors thirsty for reflection face to face with their own images, prompting a need to capture and share that moment of unexpected self-discovery. Mirrors also make it possible to show off more of the self without having to use a selfie stick (which are devices that artificially extend one’s reach and that are often prohibited in museums). Due to these qualities, museum objects that have mirrored surfaces appear very prominently in museum selfies. However, they are far less prominent in the actual, physical space of the museum than their prominence in selfies might suggest. The argument that object significance can be transferred to the self through the selfie does not, therefore, apply to these cases. One object that appears frequently in the Louvre selfies, for instance, is a baroque commode with a mirror. Clearly, what is being communicated here has less to do with the object itself or its baroque origins than with its surface. Yet, as we will see, although they like to present surfaces used to simply reflect, many museum selfies are far from superficial.

One type of performance that appears in museum selfies is the silly/clever selfie. Making funny faces or striking particularly silly poses adds individuality and makes the selfie unique, thus increasing its social media share-worthiness. It allows selfie-takers to express their personality and show off. These selfies are often accompanied by particularly clever or funny descriptions. For example, one burly, smirking young man poses in front of the Mona Lisa and captions his selfie, “One of these faces is worth one billion dollars!!” A couple posted a picture of themselves and used the caption, and its hashtags, to tell a deeper story: “Vicky and I at the Louvre. There’s a kinda famous painting behind us 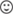 #MonaLisa #ThisMuseumIsHuge #ThePaintingIsNot”.

In a more physical form of silliness, many people pose in front of the Louvre’s pyramid with their outstretched hands, as if touching the apex of the pyramid with one fingertip. One, with a man grabbing the pyramid playfully and a young girl mugging surprise, was simply captioned “Gotcha!” In many of these photographs, selfie takers assume a performative stance, “playing to the moment” and spotlighting what is unique about the place, the object, the situation, the time, and, of course, themselves, in ways that might be otherwise difficult to perform in the “flow of routine life” (Rounds, 2006, p. 142).

We also found that other museum patrons provided selfie takers a comfortable and respectful berth when they were framing, posing, and shooting their self-portraits. Writing in a time before the rise of selfies, Rounds (2006, ibid) describes an eerily familiar scene: the museum viewer “strikes a contemplative pose” and “other patrons respond in kind, moving as if in response to an invisible choreographer, avoiding intrusions between patron and painting, signaling respect for the aesthetic experience in progress”. That such observations of general museum behavior bear such an uncanny similarity to the occurrences that happen around selfie taking in our study points to the aesthetic linkage between selfie taking and art appreciation. At the level of cultural psychological reality as embodied in the movement of people around one another in museum spaces, art and selfie seem to be intertwined.

Another type of very common performance reflected in the selfies is the performance of contemplation. These selfies show the self from behind, looking at the artwork. This selfie type is often accompanied by a more profound type of statement in the description. In one, in which the person appears as a silhouette in front of the giant transparent clock face at the Musée D’Orsay, the Instagram selfie caption reads: “Life is truly precious and I think every second becomes a privilege. Whether it be a second longer to admire a piece of art, embrace a loved one, or simply take another breath, every second becomes infinitely valuable if you recognize its worth”. Others write similarly reflective captions: “Life is made of small moments like these” “If only, sometimes, time would just stand still, in the exact moment you want it to” “Time is a storm for which we have no umbrella” “A photograph is a fragment of time that will never return”. The identity communicated through the use of such captioned selfies is introspective and rich with a sense of transcendent meaning. The spiritual type of identity work conveyed here is one of an appreciation not only of art, but of the aesthetic moments that art brings and of the precious, transitory pleasures of life itself. These contemplative and beautiful posts, where faces are indistinct or absent and captions seek to capture and communicate universal truths to a potentially limitless audience, seem far from the extant stereotype of the superficial and narcissistic selfie.

Performances of the self in museums seem to involve concentric circles of stages, from the micro-stage of the object to the exhibit to the museum space itself and even beyond, with many selfies being taken outside of museums. As observed by Hromack (2014), selfie-taking assigns greater significance to gallery spaces than ever before. The core of these stages is allowing the self to be the focus of the gaze. However, these gazes can be complex and the selves can be ironically positioned rather than self-centered. One photograph featured a young man with a red beard who resembles Van Gogh posing in front of the iconic artist’s self-portrait and challenging viewers in the caption to “Spot the difference”. A woman posed next to a Greek statuary encourages viewers to identify “Which one is the work of art?” Many selfies are taken in general museum areas, not featuring any particular object except the self, as if to state, more clearly than in any other selfie case: “I am in the museum, therefore I am the art”.

### Iconic Selfies

Iconic selfies suggest the strong influence of social norms and established social behaviors. Burness (2016, p. 99) celebrates the individualistic aspect of museum selfie taking, positing that it constitutes an “individual’s performance of self, [in which] identity is *performative* as a social role is selected” from a range of available roles [italics in the original]. Truly, the possibilities are endless for performance and creativity in a space that celebrates artistry and innovation. Yet our analysis of the netnographic data finds a disheartening conformist similarity and consistency in the many of the selfies taken at museums. Their similarity is based not only on the location but also on the poses taken and the perspectives portrayed. Through our netnographic analysis, which paid attention to similar hashtags, times, and places, we were able to see social mimicry enacted online. When someone took a selfie in a particular place, others often felt compelled to do exactly the same. Part of the identity work then may not only be establishing the self as unique and creating personalized narratives but, importantly, also to show that one did the museum as one is supposed to do it. One stands in line obediently at the Louvre, in order to get close to the Mona Lisa. Once close, one turns one’s camera upon oneself, frames the masterpiece in the background, and takes the selfie. Then, one moves along for the next selfie taker. Mimicry also allows one to establish the self as immersed in a social world of significance and meaning, a consocial experience in which a temporary connection is made with others, to be extended online.

From this perspective of the social self emerges the notion of the iconic museum selfie. The Mona Lisa selfie is the most prominent example of this category. Not only has it been advocated by celebrities but also extensively written and blogged about. One blogger writes: “Everyone else looked at her backward. So I did, too”. It has become so ubiquitous that selfie-takers now have to try to make it special in order to make it share-worthy, e.g., by trying to have nobody else in the selfie, which is almost impossible. Another example of an iconic selfie is taken in front of the clock in the Musée D’Orsay. Our dataset is filled with similar silhouettes of similar bodies posed in front of the famous clock. Some try to individualize with strange poses or photos shot from different angles. Most simply pose in the same manner before the giant clock which leads out to the sky.

Another example is technologically driven rather than spatially cued. One of the latest developments in the museum selfie genre is a selfie taken using the Snapchat app to swap faces with those portrayed in famous paintings. Whether extraordinary or not, the iconic selfie has to be taken in order to complete the museum visit. The sheer amount of iconic selfies appearing on Instagram for the particular museums provides a glimpse at how long the selfie-takers had to wait to be able to snap the particular picture of themselves. This fact suggests how important it must be, and how much the internal pressure to conform to standard must feel for them to include the iconic selfie in their self narratives, to perform the identity work, and to feel a sense of completion of their visit to the museum.

### Selfie-Taking as an Embodied Museum Experience

As indicated by our extensive data about performance, selfies turn museums into playgrounds. Selfies encourage physical engagement with museum objects. They involve poses, contorting the body in order to get the selfie poses right, waiting in line to get to the important work of art, walking through the museums with cameras, and walking around other visitors who are taking their selfies. Although some of this action is detectable in the posted selfies themselves, most of it was directly observed during our museum visits. Selfies encourage a certain consciousness of the body and its placement in space. Which body parts are framed as part of the selfie becomes an important decision in the selfie-taking process. The entire self, body, mind, and even spirit seem involved in the aesthetic process of selfie museum taking and sharing.

## Discussion and Conclusion

In our investigation of museum selfie taking we find reflections not only of individuals and their identities, but of consumer culture, the ways it is changing, and the ways that it destabilizes patrons, museums and art. First, the performance of selfie taking destabilizes the experience of being a body in a museum filled with art. The body is tethered to technology, holding the phone. The body bends and leans to get better angles, find more flattering light, more favorable positions. The body becomes an object to be photographed along with the art. In some sense, the body is led through the museum by a more overwhelming project than merely seeing the art in the gallery: the identity project of representing the self in the act of being-in-the-gallery and the even more important one of somehow asserting that the self is as worthy of art-like status as the art.

This phenomenon does not seem to reflect only the narcissism of the sort explored by researchers such as Fox and Rooney (2015), Sorokowski et al. (2015), and Lee and Sung (2016). Many posts did not feature the selfie poster’s face, many contained the back of the head while the person viewed or admired the art, many featured only a silhouette. The addition of a rich context and the cultural data and analysis provided by our method allows us to recontextualize the selfie back into human cultural life and propose an alternate and multidimensional view of the phenomenon as identity work and, indeed, identity work which is fragmentary and always somehow frustratingly incomplete.

Our findings reinforce past conceptions of selfies as important forms of collective communications (Molz, 2012) and a way to build, assert, and curate lasting narratives of the self (Papacharissi, 2010; Dinhopl and Gretzel, 2016) that contribute to an ongoing process of social media-assisted identity work (Rettberg, 2014). However, we extend these conceptions by showing the complexity and variety of ways that people use museum selfies as a part of their identity projects. As our many examples of museum selfie taking in action demonstrate, there is little doubt that physical spaces and objects such as sculptures and paintings, and museums themselves, are used as symbolic resources to build ongoing narratives of the self, just as brands are (Rokka and Canniford, 2016). Art works and museum spaces become props, background material, and stages upon which individuals act out the experiences that give their identity its uniqueness and their life its meaning.

Further, we find that the combination of museum, person, and selfie may amplify and complicate the ostensibly destabilizing forces of art, museums and their patrons. Notions of art viewers using art and their own aesthetic power over it to transcending the authoritarian powers of museum have a long basis in the history of art. To provide only one example, the panoramic size of Monet’s *Water Lilies* was intended by the artist to challenge the limited wall space and thus to allow viewers to contest the authority of the museum, and in some sense to breach the boundaries separating art from its audience (Ames, 1992). To use a more recent example, the *Sugar Baby* installation posed a powerful question to art patrons about their willingness to turn art into a background stage and sacrifice their own “sense of self-awareness when addressing an art object,” according to Larson (2014, p. 505). The art and the installation sought to destabilize the urge to photograph oneself against it, to “use” the gigantic Jemima figure in this way and reinforce her link to the suffering and abuse of black women. The artist and her brilliant work of art deliberately play on contemporary norms of selfie taking to destabilize power perceptions: “Walker is thus effortlessly able to prove to us that all those old power relationships from the time of slavery have not lost their sting, nor their roots in human self-centeredness. She sets up the conditions, and if you are alert, you see yourself and your reflection, and you may have learned something” (Larson, 2014, p. 506). For most people observed by Larson, however, nothing is learned.

Hence, museums are not simple places for learning about and enjoying art, they have always been contested spaces where we are goaded to realize something about ourselves. The many profound and spiritual statements about time which appear in selfies featuring the Musée D’Orsay attest to the undiluted power of art to inspire self-reflection. These museum stages therefore provide much more than an opportunity for superficial performance and conformist mimicry.

Our ethnography and especially our netnography of museum selfies tells a nuanced story about contemporary aesthetics. The practices we observe include enactment of the urge to put one’s self into the artwork, to shoot into shiny surfaces, to line up behind others and to collect the expected photographs of famous art works. Alongside the enactment of these typical urges, we also see how commonplace are attempts to subvert the art by making silly or idiosyncratic expressions in front of it. In the end, the acting out of these anti-authoritarian impulses seems like a predictable attempt to destabilize art’s authority. Networked digital technology seems poised to empower these efforts, to unleash a creative, individual, and aesthetic self as never before. Yet, for the most part, as our portrait of the conforming patron within the artistic environment attests, this creativity goes unrealized. The art, like *Sugar Baby*, remains spiritually pure, aesthetically untouched, never fully apprehended. Its surface reflection may be captured, digitized, and shared, but its true depths remain unplumbed, blurred out of focus by the cellphone’s public gaze. Patrons’ use of smartphone technology seems to change everything in the gallery, but it actually challenges nothing. Rather than the celebratory conclusion of Burness (2016, p. 115) who finds that “visitors engaging in self-representational social photography are paying the ultimate compliment to museums by weaving museum objects into their identity,” we must take a much more balanced view.

Our investigation thus broadens and develops Venkatesh and Meamber’s (2008, pp. 51–52) conception of individuals as “aesthetic subjects” who view everyday experiences and artistic products in an aesthetic sense. Complicating their rather harmonious view of the aesthetic self, we see members of the public actively at work, struggling with their identities as social and creative beings, unable to fully realize either one. Their attempts to transfer aesthetic impressions from art works to their own captured photographs of themselves are always incomplete in themselves, always only snapshots of a much longer narrative that they continuously construct on social media. This sense of needing to do something beyond being with the art is exacerbated and proliferated by the public spaces of museums, where patrons observe each other posing and getting into line before certain art works. Every museum becomes a stage, as we have seen. Every museumgoer becomes not only the “aesthetic object” (Venkatesh and Meamber, 2008, p. 52) of their own selfie, but a performer within their own documentary project of selfie taking. And yet, as Karwowski and Brzeski (2017) also find, many of them are ill-equipped to provide meaningfully creative output.

Every artwork is in some way a selfie, every photograph of course reveals its taker. It also can reveal its takers’ abilities and inabilities. Selfie taking, after all, is not merely a manifestation of the mirrored self-questing for its own sense of identity. It is also a social act, a call for connection, a response to competition, and act of mimicry. Our findings reveal how ubiquitous smartphone cameras and networking technology blur the way people understand the connection between museum art and their own self-portraiture. These technologies and behaviors allow people to use special locations, such as art galleries and museums to express aspects of themselves and borrow particular cultural meanings, such as the aesthetic sophistication of art.

At the museum, as in life, people taking their portraits, again and again, visually producing themselves and visualizing themselves as beings who exist “in a world they desire, full of people who want them and who want to watch them” (Kozinets et al., 2004, p. 670). Once upon a simpler time, many of these galleries existed to elevate art in a serious and educational spirit. Now, they are transforming under economic pressure to accommodate the playfulness of patrons. The fact that museums like the Groninger, curators like Blühm (2016), and artists like Kara Walker, the creator of *Sugar Baby*, must respond to this tension, points to the significant role of the museum selfie in the identity project of the art museum patron. In the field of psychology, and beyond, we would be wise to continue to explore the varying contexts and multidimensional aspects of this rich and powerful phenomenon, what it tells us about the notion of the self in contemporary society, and what it portends about where the sense of contemporary selfhood is heading.

## Ethics Statement

The study was exempt as the participant observations were focused on the researchers’ own experiences, only took place in public spaces and did not involve any direct interactions with subjects. The online component of the research used only publicly available posts and did not report individual data in the manuscript.

## Author Contributions

RK contributed to the theoretical framing, analysis of the data, as well as creation of the entire final document. UG and AD were responsible for the data collection and analysis. AD contributed to the literature review. UG was involved in all sections of the paper.

## Conflict of Interest Statement

The authors declare that the research was conducted in the absence of any commercial or financial relationships that could be construed as a potential conflict of interest.

## References

[B1] Adamkolo M. I. Elmi-Nur H. (2015). Communicating ‘the self’ through digital images: gender bias and mental health risks associated with selfie use on social network sites. *Glob. Media J. Malays. Ed.* 5 16–36.

[B2] Ames M. M. (1992). *Cannibal Tours and Glass Boxes: The Anthropology of Museums.* Vancouver, BC: UBC Press.

[B3] Appiah K. A. (2006). The politics of identity. *Daedalus* 135 15–22. 10.1162/daed.2006.135.4.15

[B4] Barry C. T. Doucette H. Loflin D. C. Rivera-Hudson N. Herrington L. L. (2017). “Let me take a selfie”: associations between self-photography, narcissism, and self-esteem. *Psychol. Pop. Media Cult.* 6 48 10.1037/ppm0000089

[B5] Belk R. W. (1975). Situational variables and consumer behaviour. *J. Consum. Res.* 2 157–164. 10.1086/208627

[B6] Belk R. W. (1988). Possessions and the extended self. *J. Consum. Res.* 15 139–168. 10.1086/209154

[B7] Belk R. W. (2013). Extended self in a digital world. *J. Consum. Res.* 40 477–500. 10.1086/671052

[B8] Bengry-Howell A. Wiles R. Nind M. Crow G. (2011). *A Review of the Academic Impact of Three Methodological Innovations: Netnography, Child-Led Research and Creative Research Methods.* Southampton: University of Southampton.

[B9] Bluühm A. (2016). Managing change at the Groninger Museum. *Mus. Manage. Curatorship* 31 96–101. 10.1080/09647775.2016.1139358

[B10] Bourgeois P. Hess U. (2008). The impact of social context on mimicry. *Biol. Psychol.* 77 343–352. 10.1016/j.biopsycho.2007.11.008 18164534

[B11] Burness A. (2016). “New ways of looking: self-representational social photography in museums,” in *Museums and Visitor Photography: Redefining the Visitor Experience*, ed. Stylianou-Lambert T. (Boston, MA: Museums Etc), 152–183.

[B12] Carbon C.-C. (2017). Universal principles of depicting oneself across the centuries: from renaissance self-portraits to selfie-photographs. *Front. Psychol.* 8:245 10.3389/fpsyg.2017.00245 PMC5318418 28270789

[B13] Chartrand T. L. Bargh J. A. (1999). The chameleon effect: the perception–behavior link and social interaction. *J. Pers. Soc. Psychol.* 76 893 10.1037/0022-3514.76.6.893 10402679

[B14] Dhir A. Pallesen S. Torsheim T. Andreassen C. S. (2016). Do age and gender differences exist in selfie-related behaviours? *Comput. Hum. Behav.* 63 549–555. 10.1016/j.chb.2016.05.053

[B15] Dimberg U. Thunberg M. Elmehed K. (2000). Unconscious facial reactions to emotional facial expressions. *Psychol. Sci.* 11 86–89. 10.1111/1467-9280.00221 11228851

[B16] Dinhopl A. Gretzel U. (2016). Selfie-taking as touristic looking. *Ann. Tour. Res.* 57 126–139. 10.1016/j.annals.2015.12.015

[B17] Falk J. H. (2006). An identity-centered approach to understanding museum learning. *Curator Mus. J.* 49 151–166. 10.1111/j.2151-6952.2006.tb00209.x

[B18] Foster M. (2014). Online and plugged in? *Public Hist. Rev.* 21 1–19.

[B19] Fox J. Rooney M. (2015). The Dark Triad and trait self-objectification as predictors of men’s use and self-presentation behaviors on social networking sites. *Pers. Individ. Diff.* 76 161–165. 10.1016/j.paid.2014.12.017

[B20] Goffman E. (1959). *The Presentation of Self in Everyday Life.* Garden City, NY: Anchor.

[B21] Hess A. (2015). Selfies: the selfie assemblage. *Int. J. Commun.* 9 18.

[B22] Hollenbeck C. R. Peters C. Zinkhan G. M. (2008). Retail spectacles and brand meaning: insights from a brand museum case study. *J. Retailing* 84 334–353. 10.1016/j.jretai.2008.05.003

[B23] Howard J. A. (2000). Social psychology of identities. *Annu. Rev. Sociol.* 26 367–393. 10.1146/annurev.soc.26.1.367

[B24] Hromack S. (2014). *The Museum Selfie.*Available at: http://whitney.org/WatchAndListen?play_id = 916

[B25] Iqani M. Schroeder J. E. (2015). # selfie: digital self-portraits as commodity form and consumption practice. *Consumpt. Mark. Cult.* 19 1–11.

[B26] Johnson L. Adams Becker S. Estrada V. Freeman A. (2015). *The NMC Horizon Report: 2015 Museum Edition.* Austin, TX: New Media Consortium.

[B27] Jones A. (2002). The ‘eternal return’: self-portrait photography as a technology of embodiment. *Signs* 27 947–978. 10.1086/339641

[B28] Joy A. Sherry J. F. (2003). Speaking of art as embodied imagination: a multisensory approach to understanding aesthetic experience. *J. Consum. Res.* 30 259–282. 10.1086/376802

[B29] Karwowski M. Brzeski A. (2017). Selfies and the (creative) self: a diary study. *Front. Psychol.* 8:172 10.3389/fpsyg.2017.00172 PMC5296334 28228743

[B30] Kozinets R. V. (2002). The field behind the screen: using netnography for marketing research in online communities. *J. Mark. Res.* 39 61–72.

[B31] Kozinets R. V. (2015). *Netnography: Redefined.* London: Sage 10.1002/9781118767771.wbiedcs067

[B32] Kozinets R. V. (2017). “Netnography: radical participative understanding for a networked communications society,” in *Handbook of Qualitative Research on Psychology*, eds Willig C. Stainton-Rogers W. (London: Sage), 376–382.

[B33] Kozinets R. V. Sherry J. F. DeBarry-Spence B. Duhachek A. Nuttavuthisit K. Storm D. (2002). Themed flagship brand stores in the new millennium: theory, practice, prospects. *J. Retailing* 78 17–29. 10.1016/S0022-4359(01)00063-X

[B34] Kozinets R. V. Sherry J. F. Storm D. Duhachek A. Nuttavuthisit K. DeBerry-Spence B. (2004). Ludic agency and retail spectacle. *J. Consum. Res.* 31 658–672. 10.1086/425101

[B35] Lacan J. (1977). *Ecrits, A Selection*, trans. A. Sheridan London: Tavistock.

[B36] Lakoff G. Johnson M. (1999). *Philosophy in the Flesh: The Embodied Mind and its Challenge to Western Thought.* New York, NY: Basic books.

[B37] Larsen J. Sandbye M. (2014). “The (im)mobile life of digital photographs: the case of tourist photography,” in *Digital Snaps: The New Face of Photography*, eds Larsen J. Sandbye M. (New York, NY: Palgrave-Macmillan), 25–46.

[B38] Larson K. (2014). A subtlety, or the marvelous sugar baby. *Curator* 57 505–511. 10.1111/cura.12088

[B39] Leder H. Belke B. Oeberst A. Augustin D. (2004). A model of aesthetic appreciation and aesthetic judgments. *Br. J. Psychol.* 95 489–508. 10.1348/0007126042369811 15527534

[B40] Lee J. A. Sung Y. (2016). Hide-and-seek: narcissism and “selfie”-related behavior. *Cyberpsychol. Behav. Soc. Netw.* 19 347–351. 10.1089/cyber.2015.0486 27028460

[B41] Levin A. (2014). *The Selfie in the Age of Digital Recursion. InVisible Culture, (20).*Available at: http://ivc.lib.rochester.edu/category/issues/archives/issue-20/ [accessed March 1 2017].

[B42] Markus H. Nurius P. (1986). Possible selves. *Am. Psychol.* 41 954 10.1037/0003-066x.41.9.954

[B43] McCracken G. (1987). “Advertising: meaning or information,” in *NA-Advances in Consumer Research* Vol. 14 eds Wallendorf M. Anderson P. (Provo, UT: Association of Consumer Research), 121–124.

[B44] Mick D. G. Buhl C. (1992). A meaning-based model of advertising experiences. *J. Consum. Res.* 19 317–338. 10.1086/209305

[B45] Miller P. J. Hengst J. A. Wang S. (2003). “Ethnographic methods: applications from developmental cultural psychology,” in *Qualitative Research in Psychology: Expanding Perspectives in Methodology and Design* eds Camic P. M. Rhodes J. E. Yardley L. (Washington, DC: American Psychological Association), 219–242. 10.4135/9781412986281.n109

[B46] Molz J. G. (2012). *Travel Connections: Tourism, Technology, and Togetherness in a Mobile World.* New York, NY: Routledge.

[B47] Mukherjee S. Salter P. S. Molina L. E. (2015). Museum spaces as psychological affordances: representations of immigration history and national identity. *Front. Psychol.* 6:692 10.3389/fpsyg.2015.00692 PMC4445245 26074846

[B48] Murray D. C. (2015). Notes to self: the visual culture of selfies in the age of social media. *Consumpt. Mark. Cult.* 18 490–516. 10.1080/10253866.2015.1052967

[B49] Nationalpost.com (2015). *Snap happy?: A Trip to the Selfie Museum in Manila, the Selfie Capital of the World.*Available at: http://news.nationalpost.com/arts/weekend-post/snap-happy-a-trip-to-the-selfie-museum-in-manila-the-selfie-capital-of-the-world?__lsa=79e4-1edb [accessed October 30, 2016].

[B50] Orsolini L. Papanti G. D. Francesconi G. Fabrizio S. (2015). Mind navigators of chemicals’ experimenters? A web-based description of E-psychonauts. *Cyberpsychol. Behav. Soc. Netw.* 18 296–300. 10.1089/cyber.2014.0486 25965863

[B51] Papacharissi Z. (ed.) (2010). *A Networked Self: Identity, Community, and Culture on Social Network Sites.* New York, NY: Routledge.

[B52] Pekarik A. J. Doering Z. D. Karns D. A. (1999). Exploring satisfying experiences in museums. *Curator* 42 152–173. 10.1111/j.2151-6952.1999.tb01137.x

[B53] Pounders K. Kowalczyk C. M. Stowers K. (2016). Insight into the motivation of selfie postings: impression management and self-esteem. *Eur. J. Mark.* 50 1879–1892. 10.1108/EJM-07-2015-0502

[B54] Presi C. Maehle N. Kleppe I. A. (2016). Brand selfies: consumer experiences and marketplace conversations. *Eur. J. Mark.* 50 1814–1834. 10.1108/EJM-07-2015-0492

[B55] Qiu L. Lu J. Yang S. Qu W. Zhu T. (2015). What does your selfie say about you? *Comput. Hum. Behav.* 52 443–449. 10.1016/j.chb.2015.06.032

[B56] Rettberg J. W. (2014). *Seeing Ourselves Through Technology: How we Use Selfies, Blogs and Wearable Devices to See and Shape Ourselves.* New York, NY: Palgrave Macmillan.

[B57] Rokka J. Canniford R. (2016). Heterotopian selfies: how social media destabilizes brand assemblages. *Eur. J. Mark.* 50 1789–1813. 10.1108/EJM-08-2015-0517

[B58] Rounds J. (2006). Doing identity work in museums. *Curator* 49 133–150. 10.1111/j.2151-6952.2006.tb00208.x

[B59] Schau H. J. Gilly M. C. (2003). We are what we post? Self-presentation in personal web space. *J. Consum. Res.* 30 385–404. 10.1086/378616

[B60] Schroeder J. E. (2002). *Visual Consumption.* New York, NY: Routledge.

[B61] Schroeder J. E. (2013). *Snapshot Aesthetics and the Strategic Imagination. InVisible Culture, (18).*Available at: https://tinyurl.com/k3mtwot [accessed March 1, 2017].

[B62] Senft T. M. Baym N. K. (2015). Selfies introduction∼ What does the selfie say? Investigating a global phenomenon. *Int. J. Commun.* 9 19.

[B63] Sorokowski P. Sorokowska A. Oleszkiewicz A. Frackowiak T. Huk A. Pisanski K. (2015). Selfie posting behaviours are associated with narcissism among men. *Pers. Individ. Diff.* 85 123–127. 10.1016/j.paid.2015.05.004

[B64] Sung Y. Lee J. A. Kim E. Choi S. M. (2016). Why we post selfies: understanding motivations for posting pictures of oneself. *Pers. Individ. Diff.* 97 260–265. 10.1016/j.paid.2016.03.032

[B65] Ustuner T. Holt D. B. (2007). Dominated consumer acculturation: the social construction of poor migrant women’s consumer identity projects in a Turkish squatter. *J. Consum. Res.* 34 41–56. 10.1086/513045

[B66] Venkatesh A. Meamber L. A. (2006). Arts and aesthetics: marketing and cultural production. *Mark. Theory* 6 11–39. 10.1177/1470593106061261

[B67] Venkatesh A. Meamber L. A. (2008). The aesthetics of consumption and the consumer as an aesthetic subject. *Consumpt. Mark. Cult.* 11 45–70. 10.1080/10253860701799983

[B68] Wang R. Yang F. Haigh M. M. (2017). Let me take a selfie: exploring the psychological effects of posting and viewing selfies and groupies on social media. *Telematics Inform.* 34 274–283. 10.1016/j.tele.2016.07.004

[B69] Warfield K. (2014). *Making Selfies/Making Self: Digital Subjectivities in the Selfie.*Available at: http://kora.kpu.ca/facultypub/8/ [accessed February 16, 2015].

[B70] Wendt B. (2014). *The Allure of the Selfie. Instagram and the New Self-Portrait.* Amsterdam: Notebooks.

